# Rapid detection of *tert*-butoxycarbonyl-methamphetamine by direct analysis in real time time-of-flight mass spectrometry

**DOI:** 10.1007/s11419-017-0400-y

**Published:** 2018-01-12

**Authors:** Ken-ichi Sugie, Daisuke Kurakami, Mamoru Akutsu, Koichi Saito

**Affiliations:** 1 Narcotics Control Department, Kanto-Shin’etsu Regional Bureau of Health and Welfare Ministry of Health, Labour and Welfare, 1-2-1 Kudan-Minami, Chiyoda-ku, Tokyo, 102-8309 Japan; 2 Narcotics Control Department, Tohoku Regional Bureau of Health and Welfare Ministry of Health, Labour and Welfare, 3-2-23 Honchou, Aoba, Sendai, 980-0014 Japan; 3 Department of Analytical Chemistry, Faculty of Pharmaceutical Sciences, Hoshi University, 2-4-41 Ebara, Shinagawa-ku, Tokyo, 142-8501 Japan

**Keywords:** *tert*-Butoxycarbonyl-methamphetamine (*t*-Boc-MP), “Masked” methamphetamine, Rapid detection by DART–TOF-MS, McLafferty rearrangement, Pyrolysis

## Abstract

**Purpose:**

Phenethylamines constitute the majority of drug-related arrests in Japan. Recently, the smuggling of *tert*-butoxycarbonyl (*t*-Boc)-protected phenethylamines has become of increasing concern, because of the difficult identification of these masked substances.

**Methods:**

In this study, a rapid and accurate method for the detection of *t*-Boc-methamphetamine (*t*-Boc-MP) by direct analysis in real time–time-of-flight-mass spectrometry (DART–TOF-MS) was developed. The efficiency of the method was evaluated by comparison with conventional gas chromatography–MS (GC–MS) and liquid chromatography–TOF-MS (LC–TOF-MS) techniques.

**Results:**

During GC–MS analysis of *t*-Boc-MP, MP was generated in the injection port, which can lead to an analytical error. In the LC–TOF-MS spectrum, fragment ions were detected, which were generated by McLafferty rearrangement in the ion source. On the other hand, in the DART–TOF-MS analysis of *t*-Boc-MP, pyrolysis could be suppressed by using a micro-syringe injection method, and the fragment ions generated by McLafferty rearrangement were still observed. Moreover, protonated *t*-Boc-MP could be detected.

**Conclusions:**

Hence, DART–TOF-MS provides a rapid and accurate method for the detection of *t*-Boc-MP, allowing suppression of the pyrolysis reaction and identification of both fragment ions and protonated *t*-Boc-MP. To our knowledge, this is the first report for detecting *t*-Boc-MP by MS techniques.

## Introduction

In Japan, methamphetamine (MP), amphetamine, and their salts are strictly regulated by the Stimulants Control Law, and represent the majority of drug-related arrests. MP is seized in various forms such as crystal, tablet, and powder forms, and some MP seizures have been found to contain adulterants such as sodium benzoate and sodium thiosulfate [1]. In particular, *N*-isopropylbenzylamine, which is a structural isomer of MP [2], is one of the most common adulterants. In addition, the use of masked illegal substances as a method of drug smuggling is of increasing concern. In 2004, the Australian Custom Police seized methyl-3-[3′,4′-(methylene dioxy)phenyl]-2-methyl glycidate, which upon hydrolysis and decarboxylation efficiently produced 3,4-methylenedioxyphenyl-2-propanone, a precursor of 3,4-methylenedioxymethamphetamine (MDMA) [3].

Recently, phenethylamines bearing a *tert*-butoxycarbonyl (*t*-Boc) group on the amine were seized [4–6]. The *t*-Boc group is one of the most commonly used amino-protecting groups in organic synthesis, such as peptide synthesis, because of the simple protection-deprotection procedures [7]. In general, the *t*-Boc group can be removed by treatment with a strong acid, such as trifluoroacetic acid, to give the original compound in high yield [8]. Deprotection can also be efficiently achieved by dissolution in distilled water at 100 °C within several tens of minutes, without using a strong acid [9]. In particular, seizures of *t*-Boc-protected MDMA and MP, phenethylamines containing secondary amino groups, have been reported. Namely, *t*-Boc-MDMA was found in a viscous and bright red liquid hair product by the Australian Custom Police. The substance was initially believed to be the MDMA precursor safrole, but further detailed analysis identified it as *t*-Boc-MDMA [4]. Moreover, *t*-Boc-MP mixed with a liquid detergent was seized in New Zealand [6]. In view of the simple protection-deprotection procedures, a variety of phenethylamine drugs could be easily masked by *t*-Boc protection of the amino group. However, no analytical data for *t*-Boc-protected phenethylamines is available to date, making drug detection difficult and inaccurate. Moreover, *t*-Boc-MP and *t*-Boc-MDMA are unregulated. Clearly, the objective of the amine-masking strategy would be to reduce the risk of smuggling illicit drugs by converting them into unregulated substances. Hence, in order to prevent their distribution worldwide, a rapid and accurate analytical method for the detection of *t*-Boc-masked drugs is needed to quickly provide the administrative authorities with the drug data.

Liquid chromatography–mass spectrometry (LC–MS) and gas chromatography–MS (GC–MS) are the most widely used techniques for illegal drug analysis. LC–time-of-flight-MS (LC–TOF-MS) is particularly useful for identifying unknown compounds, because it can easily detect protonated molecules and accurately measure the molecular mass [10, 11]. However, analytical techniques based on chromatographic separations, such as LC–TOF-MS and GC–MS, usually require relatively long measurement times. On the other hand, MS using a direct analysis in real time (DART) ion source developed by Cody et al. [12] is a rapid analytical method as compared to GC–MS and LC–TOF-MS. Moreover, high-resolution TOF-MS allows the estimation of chemical formulas from accurate mass data. Thus, DART–TOF-MS has been widely used as a screening technique in the field of food hygiene and forensic science [13, 14]. On the basis of these considerations, we envisioned that DART–TOF-MS would provide an efficient screening method for *t*-Boc-MP.

In this study, a rapid and accurate DART–TOF-MS screening method for *t*-Boc-MP was developed. The efficiency of the method was evaluated by comparison with GC–MS and LC–TOF-MS analyses.

## Materials and methods

### Materials

MP hydrochloride, which was provided by the Ministry of Health, Labor and Welfare (Tokyo, Japan), was used in this study. Di-*tert*-butyl dicarbonate was purchased from Tokyo Chemical Industry Co., Ltd. (Tokyo, Japan). All other reagents were of special grade from Kanto Kagaku Co., Ltd. (Tokyo, Japan). *t*-Boc-MP was synthesized according to the procedure described by Davis et al. [8] for the preparation of *t*-Boc-amine. The purity of synthesized *t*-Boc-MP was confirmed using GC–flame ionization detection (≧ 98%). The microsyringe used (capacity 10 μL, 23–26-gauge needle) was purchased from Hamilton Inc. (Reno, NV, USA).

### GC–MS conditions

#### GC–MS instrument

Agilent 7890A GC/5975C MSD system (Agilent Technologies, Santa Clara, CA, USA); column: DB-5MS (30 × 0.25-mm i.d., film thickness 0.25 μm; Agilent Technologies); inlet temperature: 250 °C; oven temperature: 2 min at 60 °C, 10 °C/min to 300 °C, and 5 min at 300 °C; transfer line temperature: 280 °C; injection volume: 1 μL; injection mode: split (20:1) or splitless; carrier gas: He (1.2 mL/min); ionization conditions: electron ionization, 70 eV, 150 °C; mass range: *m*/*z* 40–500.

### LC–TOF-MS conditions

LC–TOF-MS was used in MS^E^ mode, which allowed alternating low and high collision energies in one injection, thus providing precursor and fragment/product ion information, respectively. The following operational conditions were used.

#### LC instrument

ACQUITY UPLC instrument (Waters, Milford, MA, USA); column: ACQUITY UPLC HSS C18 column (150 × 2.1-mm i.d., particle size 1.8 μm; Waters); solvent A: 5 mM ammonium formate in water, pH 3; solvent B: 0.1% formic acid in acetonitrile; flow rate: 0.4 mL/min; elution program: 80% A/20% B (2-min hold) to 20% A/80% B (2–15 min, 8-min hold); injection volume: 1 μL; column temperature: 50 °C.

#### TOF-MS instrument

Xevo G2 QToF mass spectrometer (Waters); ion source: electrospray ionization in positive mode; ion source temperature: 150 °C; capillary and cone voltages: 830 and 40 V, respectively; collision energy function 1: 6 V; collision energy function 2: 10–40 V; mass range: *m*/*z* 50–1000; scan time: 0.2 s; lock mass: leucine enkephalin (*m*/*z* 556.2771).

### DART–TOF-MS conditions

#### DART ion source instrument

DART-SVP™ (Ionsense, Saugus, MA, USA); ionization mode: positive mode; helium gas flow: 3.5 L/min; ion source temperature: 200 °C; discharge electrode needle voltage: 3200 V.

#### Mass spectrometer

JMS-100LP AccuTOF LC-Plus (JEOL, Tokyo, Japan); orifice 1 voltage: 10 V; orifice 2 voltage: 5 V; orifice 1 temperature: 180 °C; ring lens voltage: 5 V; ion guide peak voltage: 300 V; reflectron voltage: 980 V; mass range: *m*/*z* 10–1000; internal mass number calibration: polyethylene glycol 200 and 400; sample injection method: microsyringe.

## Results and discussion

### GC–MS

#### Structural confirmation of the synthesized *t*-Boc-MP

**Fig. 1:**
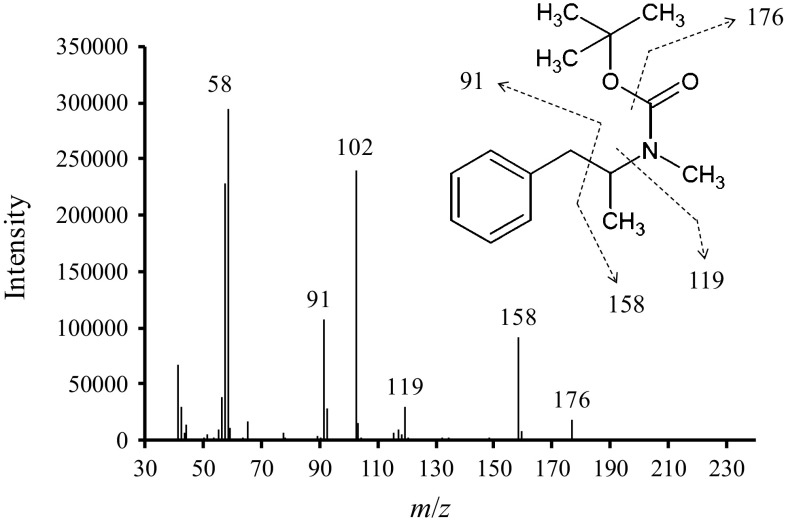
Mass spectrum of *tert*-butoxycarbonyl-methamphetamine (*t*-Boc-MP) measured by gas chromatography–mass spectrometry (GC–MS) with assigned fragmentation pattern using a split ratio of 20:1 and inlet temperature at 250 °C

The synthesized *t*-Boc-MP was analyzed by GC–MS, and the obtained mass spectrum was compared to that of *t*-Boc-MP reported by Collins et al. [4]. A solution of *t*-Boc-MP in methanol was prepared at a concentration of 100 μg/mL, and the sample was analyzed by GC–MS, using a split ratio of 20:1 and an inlet temperature at 250 °C. Notably, the mass spectrum of the synthesized *t*-Boc-MP (Fig. 1) was identical to the published one [4].

#### Pyrolysis of *t*-Boc-MP in the GC–MS injection port

**Fig. 2:**
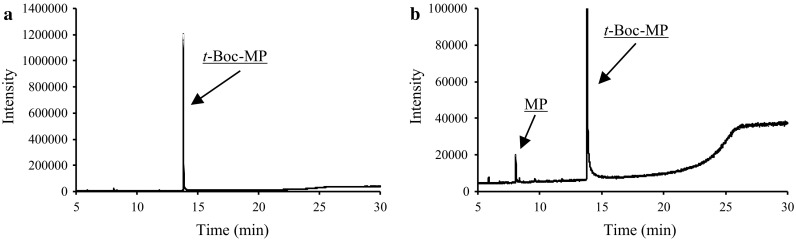
GC–MS total ion current chromatograms (TICCs) of *t*-Boc-MP at an injector temperature of 300 °C. **b** Expanded view of the chromatogram shown in **a**

MP was detected in the total ion current chromatogram (TICC) by GC–MS for the synthesized *t*-Boc-MP, and was presumably generated by pyrolysis of *t*-Boc-MP in the injection port of the instrument. In order to investigate the pyrolysis behavior of *t*-Boc-MP, GC–MS analyses were carried out at different inlet temperatures, namely, 200, 220, 250, and 300 °C, and at a split ratio of 20:1. The chromatogram recorded at an inlet temperature of 300 °C is shown in Fig. 2, and the MP-to-*t*-Boc-MP peak area ratios and *t*-Boc-MP peak areas at each inlet temperature are summarized in Table 1. MP was detected at 250 and 300 °C, but not at 200 and 220 °C. Notably, the MP-to-*t*-Boc-MP peak area ratio increased with increasing inlet temperature, whereas the *t*-Boc-MP peak area decreased. Next, the effect of the injection mode on the pyrolysis of *t*-Boc-MP was investigated, and the results are summarized in Table 2. The injector was operated in splitless and split modes (namely, 10:1, 20:1, and 50:1), while the inlet temperature was set to 200 °C. MP was detected when the GC was operated at a 10:1 split ratio and in splitless mode, but not at split ratios of 50:1 and 20:1. In splitless injection, the *t*-Boc-MP peak area was, of course, larger than that obtained using a split injection mode, suggesting that the pyrolysis of *t*-Boc-MP in the injection port was promoted at lower split ratios, which are associated with a longer heating time. These results confirmed that MP was generated by pyrolysis of *t*-Boc-MP in the injection port during GC–MS analysis. Hence, specific GC–MS conditions can lead to the formation of MP with the risk of incorrect analytical results.

**Table 1:** Methamphetamine (MP)-to-*tert*-butoxycarbonyl-methamphetamine (*t*-Boc-MP) peak area ratios and *t*-Boc-MP peak areas measured by gas chromatography–mass spectrometry (GC–MS) at different inlet temperatures with split injection at 20:1

Inlet temperature (°C)	Peak area ratio (MP-to-*t*-Boc-MP; ± SD, *n* = 3)	*t*-Boc-MP area (× 10^7^, ± SD, *n* = 3)
200	0	3.7 ± 0.2
220	0	3.0 ± 0.1
250	0.0022 ± 0.000049	2.6 ± 0.3
300	0.0145 ± 0.00061	2.3 ± 0.7

*SD* standard deviation

**Table 2:** MP-to-*t*-Boc-MP peak area ratios and *t*-Boc-MP peak areas measured by GC–MS for each injection mode at inlet temperature of 200 °C

Injection mode	Peak area ratio (MP-to-*t*-Boc-MP; ±SD, *n* = 3)	*t*-Boc-MP area (× 10^7^, ± SD, *n* = 3)
Splitless	0.012 ± 0.00034	80.2 ± 0.06
Split (10:1)	0.0024 ± 0.00017	5.27 ± 0.01
Split (20:1)	0	3.70 ± 0.21
Split (50:1)	0	0.67 ± 0.02

### LC–TOF-MS

**Fig. 3:**
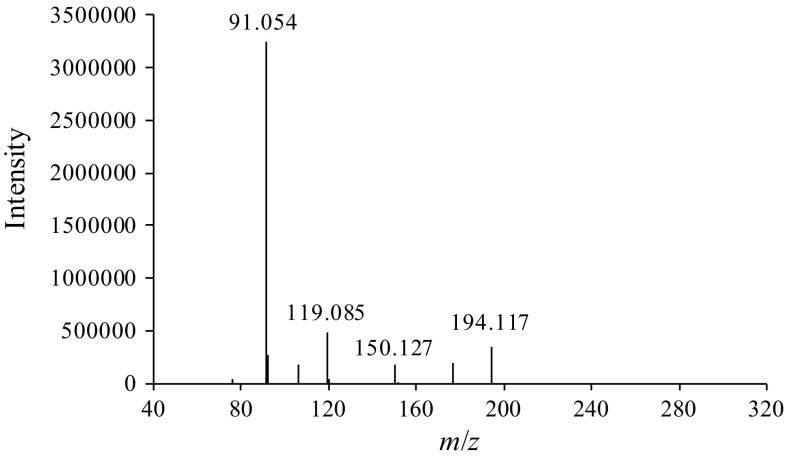
Mass spectrum of *t*-Boc-MP measured by liquid chromatography–time-of-flight-mass spectrometry (LC–TOF-MS)

**Fig. 4:**
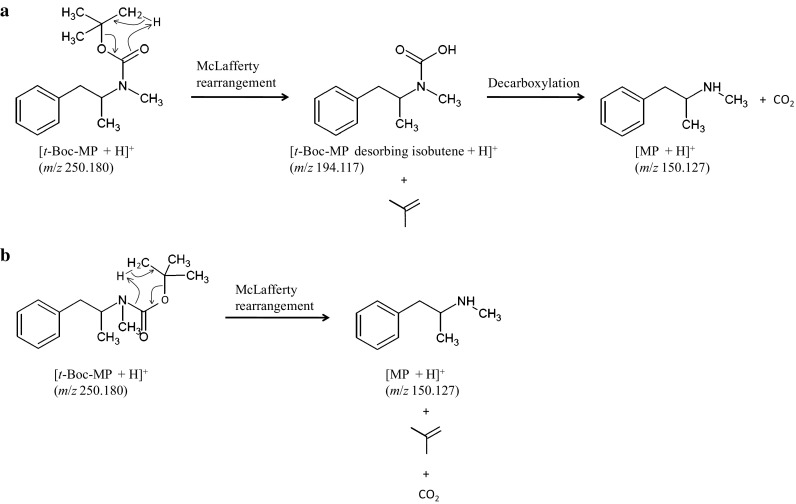
Possible pathways for the McLafferty rearrangement of *t*-Boc-MP during LC–TOF-MS analysis: **a** pathway producing an intermediate derived from isobutene desorption (*m*/*z* 194.117) and methamphetamine (MP) (*m*/*z* 150.127); **b** pathway producing only MP (*m*/*z* 150.127). All *m*/*z* values refer to the corresponding protonated compounds

A 100-ng/mL methanol solution of *t*-Boc-MP was prepared and analyzed by LC–TOF-MS. As can be seen from the mass spectrum in Fig. 3, the ion at *m*/*z* 91.054 was detected as the base peak, and fragment ion peaks at *m*/*z* 119.085, 150.127, and 194.117 were also observed. However, protonated *t*-Boc-MP (theoretical *m*/*z* value: 250.180) was not detected. According to the report of Wolf et al. [15], *t*-Boc-protected amines undergo McLafferty rearrangement in the ion source of the LC–MS instrument. Thus, a similar rearrangement of *t*-Boc-MP was expected to occur during LC–TOF-MS analysis, and the two previously described pathways [15] were considered. In one pathway, isobutene desorption generates an intermediate (*m/z* 194.117), which undergoes decarboxylation to produce MP (*m/z* 150.127; Fig. 4a). In the second pathway, isobutene desorption and decarboxylation occur simultaneously to yield MP directly (Fig. 4b). In our study, both fragment ions at *m*/*z* 150.127 and 194.117 were detected, indicating that the McLafferty rearrangement of *t*-Boc-MP proceeded via the pathway shown in Fig. 4a. Hence, although it was difficult to detect the protonated molecular ion of *t*-Boc-MP by LC–TOF-MS, detection of the fragment ions derived from the McLafferty rearrangement allows the identification of *t*-Boc-MP.

### DART–TOF-MS

#### Comparison of sample injection methods

**Fig. 5:**
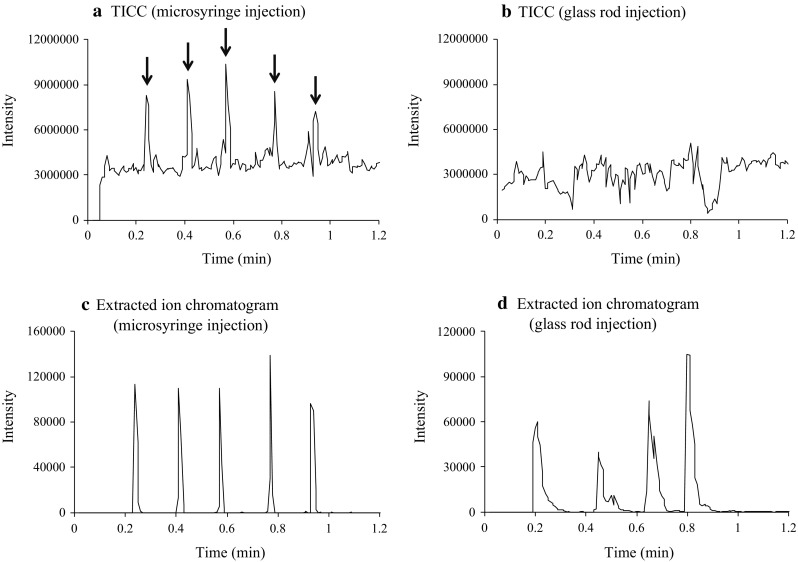
Comparison between microsyringe and glass rod injection methods in the direct analysis in real time–time-of-flight-mass spectrometry (DART–TOF-MS) analysis of *t*-Boc-MP (100 μg/mL in methanol): **a** TICC obtained using a microsyringe, **b** TICC obtained using a glass rod, **c** extracted ion chromatogram (*m*/*z* 250.180) obtained from **a**, **d** extracted ion chromatogram (*m*/*z* 250.180) obtained from **b**. The *black arrows* in **a** indicate the peaks of *t*-Boc-MP

A glass rod is commonly used as a sample injection device for DART–TOF-MS; however, the sample peak shape degraded into split peaks in the chromatogram. In order to overcome this problem, a microsyringe injection method was previously devised and applied to the quantitative analysis of new psychoactive substances including α-pyrrolidinovalerophenone by DART–TOF-MS [16]. Thus, the effectiveness of the microsyringe injection method for DART–TOF-MS analysis of *t*-Boc-MP was examined in this study. A 100-μg/mL methanol solution of *t*-Boc-MP was analyzed by DART–TOF-MS at an ion source temperature of 250 °C using a glass rod or a microsyringe as a sample injection method [16]. The TICCs and extracted ion chromatograms of protonated *t*-Boc-MP (*m/z* 250.180) obtained from the corresponding TICC are shown in Fig. 5. Using the microsyringe injection technique, distinct peaks could be identified in TICC (Fig. 5a), which were confirmed by sharp peaks in the extracted ion chromatogram (Fig. 5c). On the contrary, using a glass rod for sample injection, individual peaks were not resolved in TICC (Fig. 5b), but could be observed in the corresponding extracted ion chromatogram (Fig. 5d). However, deterioration of the peak shape was observed due to tailing (Fig. 5d). These results suggest that the microsyringe injection method was more suitable for sensitive detection of *t*-Boc-MP by DART–TOF-MS.

#### Effect of solvent

**Fig. 6:**
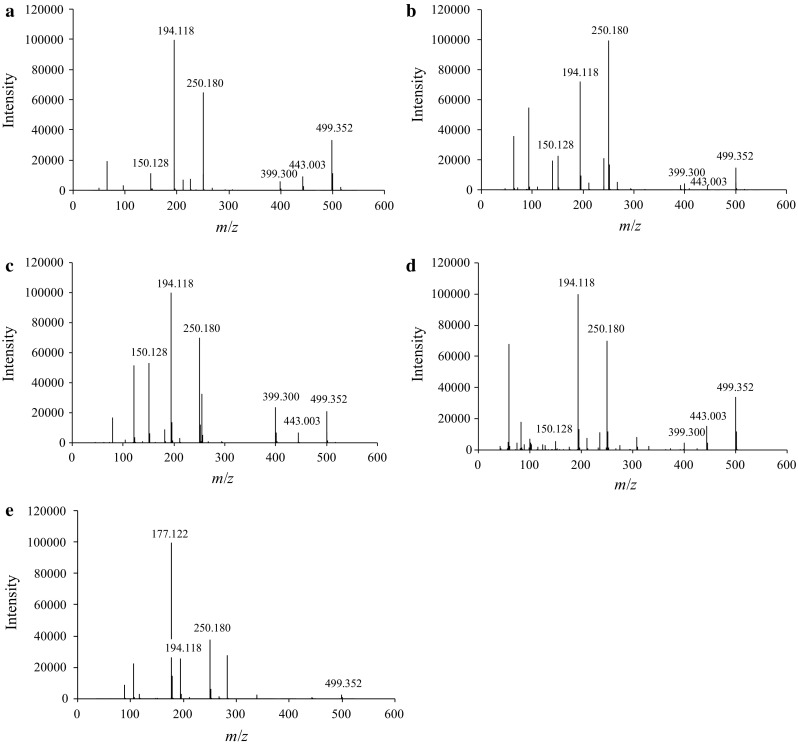
Comparison of DART–TOF-MS spectra of *t*-Boc-MP (100 μg/mL) in different solvents: **a** methanol, **b** ethanol, **c** isopropanol, **d** acetonitrile, and **e** ethyl acetate solutions. The ion source temperature was set at 200 °C

In order to determine the best solvent for DART–TOF-MS analysis, 100-μg/mL *t*-Boc-MP solutions were prepared in different solvents, namely, methanol, ethanol, isopropanol, acetonitrile, and ethyl acetate. All samples were analyzed at an ion source temperature of 200 °C. Notably, protonated *t*-Boc-MP (*m*/*z* 250.180) was detected in all solutions (Fig. 6), whereas protonated MP (*m*/*z* 150.128) was detected in all solvents except for the ethyl acetate solution. Moreover, the protonated isobutene-desorbed *t*-Boc-MP (*m*/*z* 194.118; Fig. 4a) was found in all solutions. This suggests that *t*-Boc-MP underwent the McLafferty rearrangement also during DART–TOF-MS analysis. Moreover, unlike the case of GC–MS, pyrolysis was not observed in the DART–TOF-MS measurements, irrespective of the solvent used.

In addition, all spectra showed a peak at *m*/*z* 499.352, which was attributed to the dimer of *t*-Boc-MP, whereas the peaks at *m*/*z* 399.300 and 443.003, corresponding to the dimers of *t*-Boc-MP with MP and with isobutene-desorbed *t*-Boc-MP, respectively, were detected in all spectra except for the ethyl acetate sample. However, in the case of the ethyl acetate sample, the most intense peak was found at *m*/*z* 177.122, which corresponded to the dimer form of ethyl acetate. Moreover, the impurity peaks related to the solvent and water molecules were detected in the mass range below *m*/*z* 150.128 in all solvent samples. The impurity peaks related to methanol had lower ionic strengths as compared to those for the other solvents. Hence, in this respect, methanol proved to be the best solvent for DART–TOF-MS analysis of *t*-Boc-MP.

#### Effect of ion source temperature

**Fig. 7:**
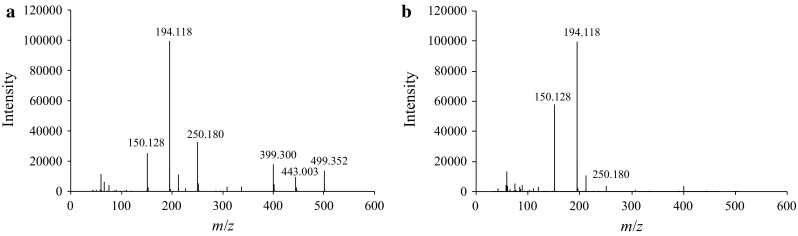
DART–TOF-MS spectra of *t*-Boc-MP. DART ion source temperatures: **a** 250 °C, **b** 300 °C

The influence of the DART ion source temperature was investigated by analyzing the *t*-Boc-MP methanol solution at 200, 250, and 300 °C. As a result, the peak of protonated *t*-Boc-MP was detected also at ion source temperatures of 250 and 300 °C, with lower intensity as compared to that of the spectrum at 200 °C. On the other hand, protonated dimers were almost not detected at an ion source temperature of 300 °C (Fig. 7). Moreover, the intensity of the MP ion (*m*/*z* 150.128) generated by McLafferty rearrangement increased with increasing ion source temperature (Figs. 6a, 7b). According to Harris et al. [17], the internal energy of analytes increases with increasing DART ion source temperature. This increase in internal energy can explain the increased formation of MP by McLafferty rearrangement and the lower formation of *t*-Boc-MP and dimeric species. Hence, an ion source temperature at 200 °C was more suitable for reliable detection of protonated *t*-Boc-MP and fragment ions by DART–TOF-MS analysis.

#### Effect of sample concentration

**Fig. 8:**
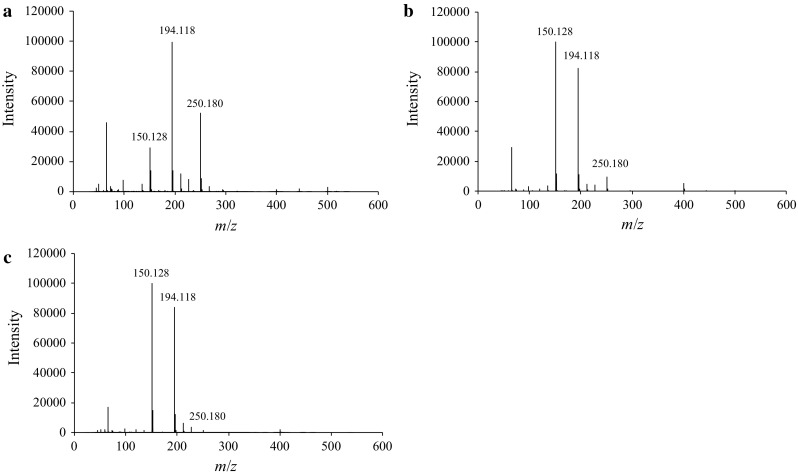
DART–TOF-MS spectra of *t*-Boc-MP at a lower concentration (10 μg/mL). DART ion source temperatures: **a** 200, **b** 250, and **c** 300 °C

Accurate identification of *t*-Boc-MP by DART–TOF-MS analysis could be achieved by the detection of not only protonated *t*-Boc-MP but also the fragment ions generated from the McLafferty rearrangement, which could be improved by suppressing the protonated dimer peaks. Because the dimers were formed at a high *t*-Boc-MP concentration of 100 μg/mL, the effect of sample concentration on the DART–TOF-MS measurement was examined by analyzing a low concentration (10-μg/mL) of *t*-Boc-MP in methanol. Notably, the dimeric species were not detected at ion source temperatures of 200, 250, and 300 °C; however, the intensity of the protonated *t*-Boc-MP ion peak decreased at ion source temperatures of 250 and 300 °C (Fig. 8). Moreover, using an ion source temperature of 200 °C, the intensity of *m*/*z* 150.128 was higher at a concentration of 10 μg/mL than at 100 μg/mL (Fig. 6a), and a relatively high intensity of the protonated *t*-Boc-MP peak was observed. These results show that the formation of dimers can be suppressed by using a diluted sample, which allows highly sensitive detection of fragment ions and protonated *t*-Boc-MP by DART–TOF-MS.

## Conclusions

In this study, a DART–TOF-MS method was developed for the rapid and accurate detection of *t*-Boc-MP by using a microsyringe injection technique, methanol as a solvent, an ion source temperature of 200 °C, and a sample concentration of 10 μg/mL. This method, as a screening test, proved to be superior to GC–MS and LC–TOF-MS analyses. During GC–MS analysis, *t*-Boc-MP underwent pyrolysis in the injection port under certain operational conditions, which can lead to an analytical error. In the LC–TOF-MS spectrum, ion fragments generated by McLafferty rearrangement were detected, whereas the protonated *t*-Boc-MP parent ion was not found. On the other hand, in the DART–TOF-MS analysis, pyrolysis was not observed with any of the solvents. Moreover, similarly to LC–TOF-MS, in the DART–TOF-MS spectrum, the fragment ions formed by McLafferty rearrangement were detected, as well as the protonated *t*-Boc-MP peak. Hence, DART–TOF-MS provides a rapid and accurate method for the detection of *t*-Boc-MP with significant reduction of the analytical error by suppression of the pyrolysis reaction, and for the identification not only with fragment ions but also with the protonated *t*-Boc-MP.  To our knowledge, this is the first report dealing with detection of *t*-Boc-MP by MS techniques.
